# Study on the Bonding Performance of Reinforced Concrete with Reef Limestone Under the Combined Effects of Dry and Wet Carbonation

**DOI:** 10.3390/ma18091963

**Published:** 2025-04-25

**Authors:** Yiyang Xiong, Fei Meng, Dengxing Qu, Mingju Mao, Jinrui Zhang

**Affiliations:** 1 School of Civil Engineering and Architecture, Wuhan University of Technology, Wuhan 430070, China307581@whut.edu.cn (D.Q.);; 2 Sanya Science and Education Innovation Park, Wuhan University of Technology, Sanya 572024, China

**Keywords:** dry–wet cycle, concrete, center pull-out test, bonding strength

## Abstract

To elucidate the mechanism underlying the changes in the bonding performance of reinforced reef limestone concrete under dry–wet carbonation cycles, and to establish a foundation for its durability analysis and design, experiments were conducted with varying dry–wet carbonation cycles (0, 20, 40, 60, and 80 cycles) and loading rates (0.01 mm/min, 0.1 mm/min, 1 mm/min, 2 mm/min, and 5 mm/min) through pull-out tests. The results demonstrate that as the number of dry–wet carbonation cycles increases, the damage to reinforced reef limestone concrete intensifies progressively, reaching a mass loss rate of 3.05% by the end of the cycles, while the ultrasonic wave velocity decreases by 17.4%. The effects of different loading rates and cycle counts on reinforced reef limestone concrete are primarily observed through alterations in peak bond stress. Utilizing the experimental data, this study established an equation to analyze the influence of dry–wet carbonation cycles and loading rates on the bond strength and slip behavior between steel bars and reef limestone concrete. This equation offers a theoretical framework for the durability analysis and design of reinforced reef limestone concrete.

## 1. Introduction

With the rapid development of the economy and technology, the scale of human exploitation and utilization of marine resources has been increasingly expanding. In recent years, numerous marine engineering projects have been constructed in our country, such as ports and docks, cross-sea bridges, offshore wind power, and drilling platforms [1,2,3]. These projects are primarily based on concrete structures that have been exposed for extended periods to harsh environments characterized by high humidity, high salinity, prolonged sunlight, and typhoons, resulting in increasingly noticeable durability issues.

In the application of concrete structures for marine engineering, due to the difficulty in obtaining terrestrial construction materials during the prolonged construction period in offshore areas, reef limestone is selected as an aggregate for concrete sourced on-site. When the prepared reef limestone concrete is used in marine environments, the porous structure of the reef limestone coarse aggregate can provide channels for the permeation of corrosive ions from seawater into the concrete, making the reinforcing steel within the reef limestone more susceptible to corrosion [4,5,6]. Kakooei S and Akil H. M et al. [7] studied the permeability characteristics of oxygen in concrete and its impact on the corrosion of internal reinforcement. They found that coral concrete, with the same mix ratio and curing time of 28 days as ordinary concrete, exhibited a reinforcement corrosion rate approximately three times higher than that of ordinary concrete. During the immersion state, corrosive ions enter the structure through the pores, and during the drying process, unreacted ions crystallize out. In this process, the pore size of concrete and environmental factors significantly affect the deterioration of the concrete under wet–dry cycles. The crystallization of salts during wet–dry cycles further exacerbates the deterioration of the concrete. When concrete is in this state, corrosive substances can corrode the concrete at a relatively rapid rate due to the coupling effects of multiple fields. The corrosion rate of concrete is 3–10 times faster underwater [8,9]. Luo Daming et al. [10] studied the degradation behavior of concrete in high salt environments. Research has found that the failure of concrete accelerates rapidly in high temperatures and humid environments. The main damage features include spalling of the concrete cover and exposure of the steel bars.

Particularly, the reef limestone concrete located in the tidal and splash zones is subjected to a long-term environment of wetting and drying cycles [11]. Under these conditions, the deterioration of concrete due to crystallization is primarily caused by the crystallization of sulfates [12,13]. The mechanisms involved include the crystallization pressure generated when Na_2_SO_4_ crystals transform into Na_2_SO_4_·10H_2_O, the hydration pressure arising from the hydration process of sodium sulfate, and the crystallization pressure resulting from sodium sulfate becoming oversaturated. When there are different concentrations of sulfates, the erosion products vary significantly [14]. In addition, the concrete in tidal and splash zones is not only subjected to wetting and drying cycles but also influenced by carbon dioxide. The concentration of carbon dioxide can accelerate the deterioration of reinforced concrete [15]. Many studies have shown a close relationship between concrete carbonation and the corrosion of reinforcement steel [16]. During the hydration of cement, numerous micropores and air bubbles are formed. It is these micropores that allow carbon dioxide to penetrate the concrete. As a result, a reaction occurs with the cement in the pores, producing calcium carbonate. The chemical reactions for concrete carbonation are as follows [17,18]:

(1) $$CO_{2}+H_{2}O\to H_{2}CO_{3}$$

(2) $$Ca{\left(OH\right)}_{2}+H_{2}{CO}_{3}\to {CaCO}_{3}+2H_{2}O$$

(3) $$3CaO\cdot 2{SiO}_{2}\cdot 3H_{2}O+3H_{2}CO_{3}\to 3CaCO_{3}+2{SiO}_{2}+6H_{2}O$$

(4) $$2CaO\cdot SiO_{2}\cdot 4H_{2}O+2H_{2}CO_{3}\to 2CaCO_{3}+{SiO}_{2}+6H_{2}O$$

After carbonation, concrete generates calcium carbonate, leading to a decrease in the pH value of concrete. After complete carbonation, the pH value of concrete is approximately 8.5 to 9.0. At this point, the reinforcing steel begins to rust, resulting in reduced durability. Karimi, Amir, et al. [19] studied the factors influencing concrete carbonation and found that carbonation is mainly affected by humidity and temperature. Ting Du [20] specifically studied the relationship between the rate of concrete carbonation and temperature, concluding that the carbonation rate has a parabolic relationship with humidity.

During the service life of reef limestone concrete structures, they are subjected to the dual effects of dry–wet carbonation for a long time, which reduces the service life of the structure and even causes irreversible disasters. At present, there are many studies on the bonding performance of steel bars and ordinary concrete, but there are not many studies on the bonding performance of reinforced reef limestone concrete under dry–wet carbonation cycles at different loading rates. Therefore, the research in this paper can provide more effective solutions for projects in island areas and ensure the long-term safety and stability of the projects.

## 2. Experimental Overview

### 2.1. Preparation of Test Pieces

Mix the reef limestone coarse and fine aggregates with ordinary Portland cement, water, and additives (fly ash and slag) in the proportions indicated in Table 1 to prepare concrete with a strength of C30 (physical properties are shown in Table 2). Prior to mixing the reef limestone concrete, the reef limestone aggregates are soaked in a water-filled container for 12 h to remove harmful ions and impurities. Additionally, to reduce concrete shrinkage and cracking and to improve the impermeability and durability of concrete, a polycarboxylic acid superplasticizer is added during the mixing process. The blended mixture is then placed into molds for compaction. After 24 h, the molds are removed, and the demolded reef limestone concrete samples are cured in a standard curing laboratory for 28 days. 

The reinforcement bars used are ribbed steel bars of grade HRB400 (Yede Yi Building Materials, Suzhou, China). According to the testing requirements, they are processed into several short bars with a length of 400 mm using a rebar cutting machine for future use. Reinforcement parameters are shown in Table 3.

**Figure 1:**
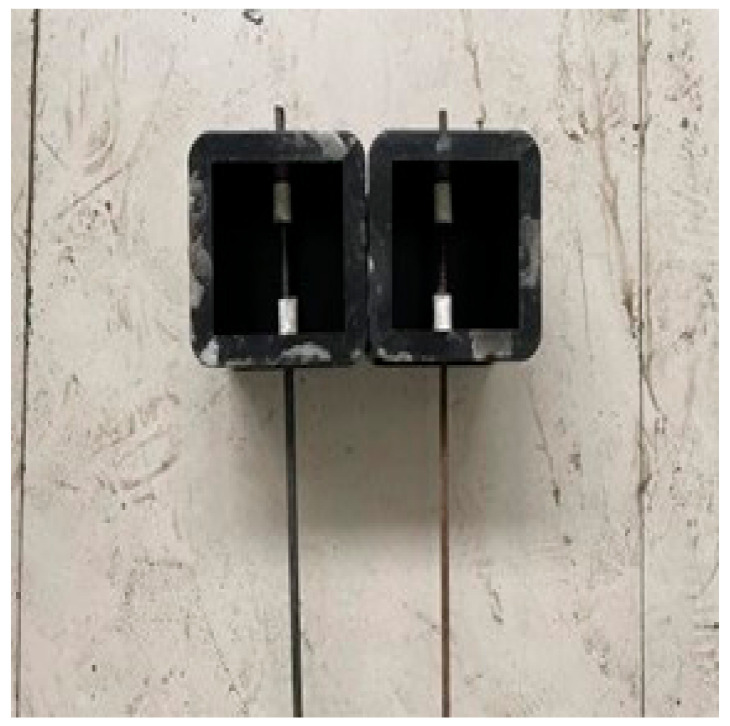
Bonding Specimen Mold.

For the specimen preparation, plastic molds with an internal edge length of 100 mm are selected. Small electric drills are used to drill holes at the center position on both sides of the mold, with hole diameters greater than that of the rebar, ensuring the embedded rebar is aligned on the same axis. The drilling diameter is based on the diameter of the rebar. PVC sleeves are then bonded with epoxy resin at the non-bonded locations. The rebar is placed into the mold according to the designed dimensions, as shown in Figure 1. Concrete is poured into the molds based on the mix ratio of the reef limestone concrete specified in Table 1. After pouring the concrete into the molds, it is placed on a vibration table and vibrated until no bubbles are visible on the surface, at which point the vibration is stopped and the surface is smoothed. After 24 h, the molds are removed, and the specimens are placed in a standard curing room for 28 days.

### 2.2. Anchorage Dry–Wet Carbonation Cycle Test

The seawater used in the test is made according to the specifications of ASTM D1141-98, and its chemical composition is shown in Table 4 [21]. The dry–wet carbonation cycle regime for this experiment is determined as follows in accordance with GB/T50082-2009 “Standard for Test Methods of Long-term Performance and Durability of Ordinary Concrete”:

(1) The specimens are immersed in a seawater solution, with the solution level at least 20 mm above the top surface of the specimens. The immersion period from the moment the specimens are placed in the solution until the end of the soaking process should be 2 days. The test solution is replaced monthly, and the temperature of the solution is controlled at (25–30) °C.

(2) After the immersion process in the seawater solution is completed, the surface moisture of the reef limestone concrete specimens is wiped dry, and the specimens are placed in a ventilated area to air dry. Subsequently, the specimens are dried in an oven. The temperature is raised from 0 degrees to 50 degrees within 30 min, and once the temperature reaches 50 °C, it is maintained at that temperature. The duration from the start of heating to the initiation of cooling for the specimens is 2 days.

(3) Once the drying of the specimens is completed, they are cooled to room temperature and then placed in a carbonation chamber. The carbonation conditions are set at a temperature of 20–30 °C, relative humidity of 70% ± 10%, and CO_2_ concentration of 20% ± 3%. Rapid carbonation is conducted, and the specimens are removed after 2 days of carbonation.

(4) The experimental regimen consists of a cycle lasting 6 days, with total cycle counts of 20, 40, 60, and 80 times. At the end of every two carbonation cycles, the mass, wave velocity, and carbonation depth of the specimens are measured.

(5) Carbonation depth testing: After the specimens are split using a compression testing machine, the residual powder on the fractured surface is scraped away. A 1% phenolphthalein alcohol solution is applied drop by drop using a pipette. After 30 s, the carbonation depth is measured at intervals of 10 mm along the fracture surface using a caliper, and the average carbonation depth of all measurement points is taken as the measured carbonation depth for that specimen.

(6) This experiment mainly studies the effect of the loading rate on the bonding performance of reinforced reef limestone concrete under dry–wet carbonation cycles. The test plan designs five different dry–wet carbonation cycles: 0, 20, 40, 60, and 80 times. For each cycle, five different pull-out rates are set: 0.01 mm/min, 0.1 mm/min, 1 mm/min, 2 mm/min, and 5 mm/min. The specimen number is identified according to the number of cycles and loading rate. For example, P-0-0.01 means that the loading rate is 0.01 mm/min under zero cycles.

During the carbonation process of the reef limestone concrete specimens, it is necessary to check the carbonation depth at regular intervals. The detection of carbonation depth during the carbonation process mainly uses the phenolphthalein reagent method to assess the carbonation depth of the reef limestone concrete. The testing is conducted through the following method: after the reef limestone concrete specimens have undergone a certain stage of carbonation, they are cut to observe the carbonation depth. The cutting direction is perpendicular to the direction of the reinforcing bars in the reef limestone concrete. After cutting, the cross-section is cleaned, and then, after natural air drying, a 1% alcohol phenolphthalein reagent is sprayed on it.

**Figure 2:**
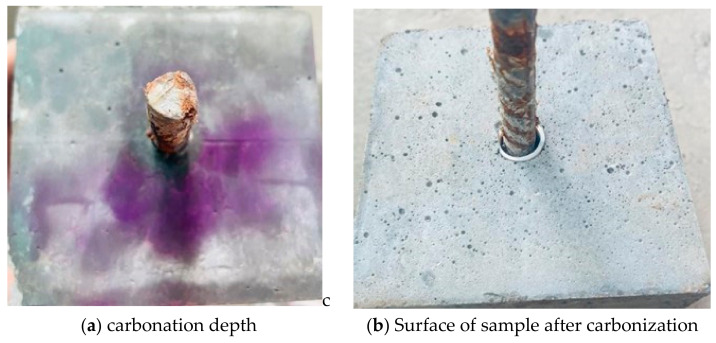
Carbonation of Reinforced Reef Limestone Concrete Specimens.

Uncarbonated reef limestone concrete appears purple–red, while carbonated reef limestone concrete is colorless. As shown in the figure below, the uncarbonated parts of the reef limestone concrete exhibit purple–red coloration, whereas the carbonated parts are colorless. The figure also shows that carbonation has reached the surface of the reinforcing bars, indicating that it is essentially fully carbonated. The phenolphthalein test results are as shown in Figure 2, meeting the testing requirements.

### 2.3. Pull-Out Test of Anchorage

#### 2.3.1. Test Scheme

**Figure 3:**
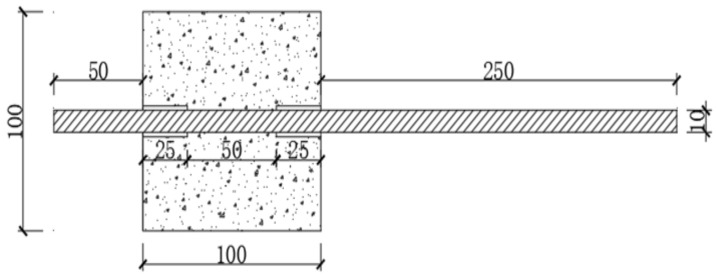
Detailed Structure of Central Pull-out Specimen.

This study uses central pull-out specimens designed according to the requirements of the Canadian Standards Association (CSA). The designed dimensions are 100 mm × 100 mm × 100 mm. To avoid damage to the reinforcing bars caused by stress concentration in the concrete near the loading end, the bonded segment of the rebar is set at the free end, with the loading end set at the free segment. PVC sleeves are used to isolate the rebar from the reef limestone concrete. The bonding performance of the reef limestone concrete under the coupled effect of dry–wet carbonation is studied using central pull-out specimens with a bonding length of 50 mm. A detailed diagram of the bonding specimen structure is shown in Figure 3.

The bonding specimen employs the central pull-out method, using cube specimens with an edge length of 100 mm. A 10 mm diameter HRB400 hot-rolled ribbed steel bar (Yedeyi Building Materials, Suzhou, China) is embedded along the central axis, with a 25 mm-long unbonded segment arranged at both the anchorage loading end and the free end. The unbonded portions are isolated using PVC sleeves, ensuring that the final bonding length of the steel bar within the reef limestone concrete reaches 50 mm. A 50 mm length of rebar is reserved at the free end for attaching a displacement gauge to measure the slip at the free end. A 250 mm length of rebar is reserved at the loading end to apply the load.

This experiment mainly investigates the effect of loading rate on the bonding performance of the anchorage of the rebar in reef limestone concrete under dry–wet carbonation cycle conditions. When the reinforced concrete is subjected to different loads, the effects of the loading rate differ in various parts of the structure. The number of dry–wet carbonation cycles refers to the scheme designed in Table 5, with carbonation at 0, 20, 40, 60, and 80 cycles. The loading rates for the specimens are designed as 0.01 mm/min, 0.1 mm/min, 1 mm/min, 2 mm/min, and 5 mm/min. Each condition group consists of three specimens. The specimen numbers are designated based on the number of cycles and loading rates; for example, P-0-0.1 indicates a specimen under zero cycles with a loading rate of 0.1 mm/min.

#### 2.3.2. Equipment

**Figure 4:**
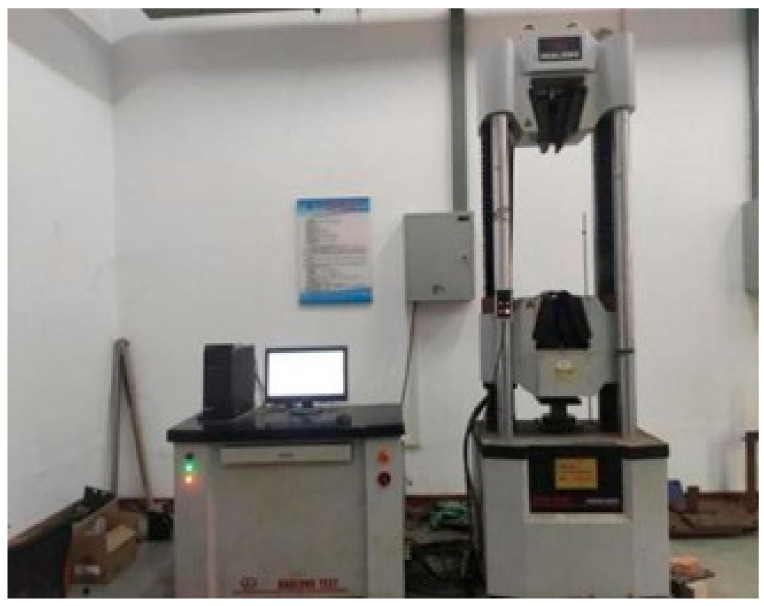
WAW-1000 Electro-hydraulic Servo Universal Testing Machine.

The central pull-out test was mainly conducted in the structural laboratory of Wuhan University of Technology, using a WAW-100 microcomputer-controlled electro-hydraulic servo universal testing machine produced by Shanghai Hualong Company (Shanghai, China), as shown in Figure 4.

**Figure 5:**
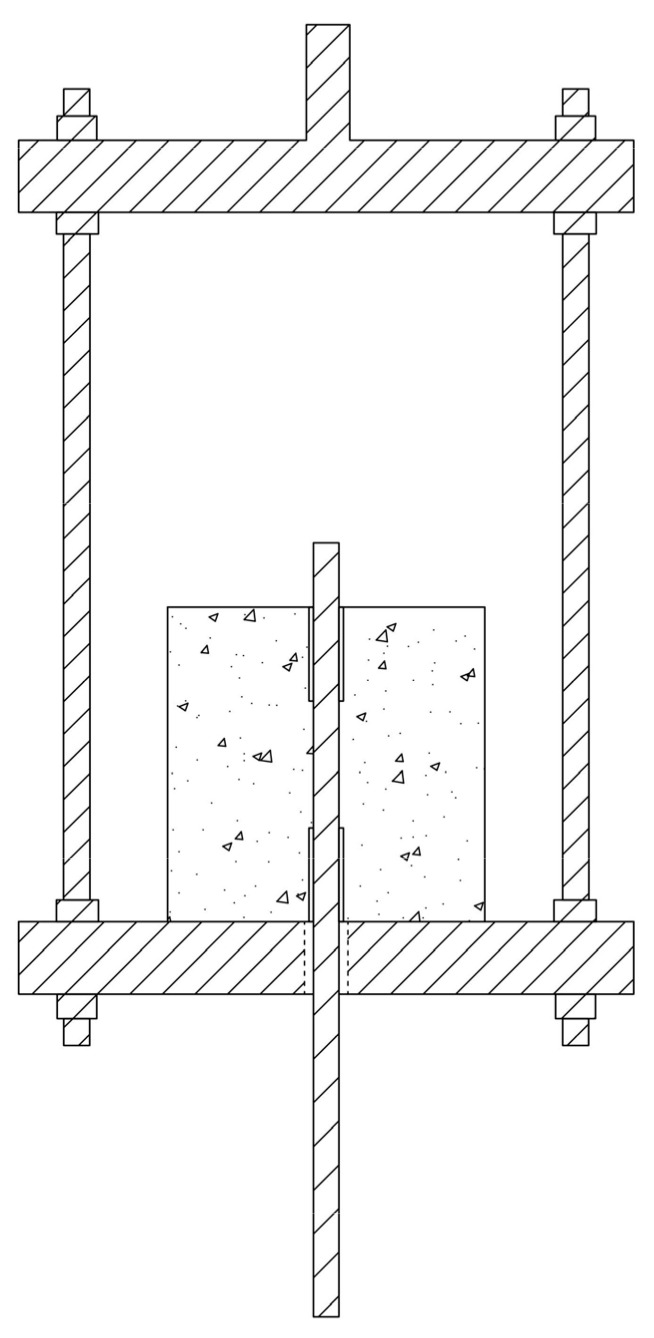
Schematic Diagram of Loading Frame.

Since the universal testing machine is primarily designed for tensile and compressive testing of specimens, a loading frame device also needs to be designed for the central pull-out test, as shown in Figure 5.

**Figure 6:**
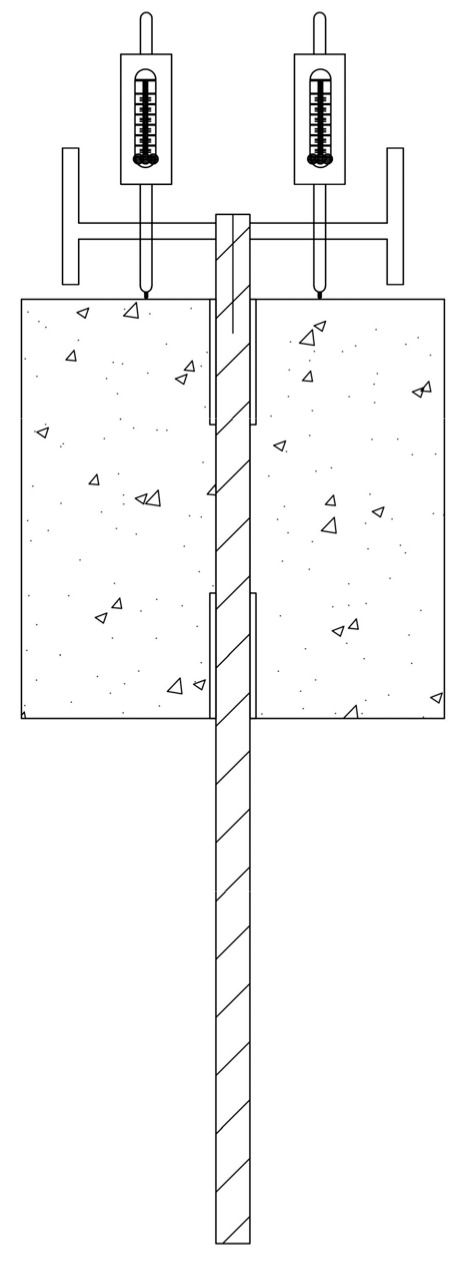
Schematic Diagram of Displacement Sensor Arrangement.

To establish the load–displacement relationship curve at the interface between the reinforcing bars and the reef limestone concrete, it is necessary to measure the relative slip between the ribbed steel bars and the reef limestone concrete. Therefore, a high-precision YHD-50 displacement sensor is installed on both sides of the free end of the rebar, as shown in Figure 6.

**Figure 7:**
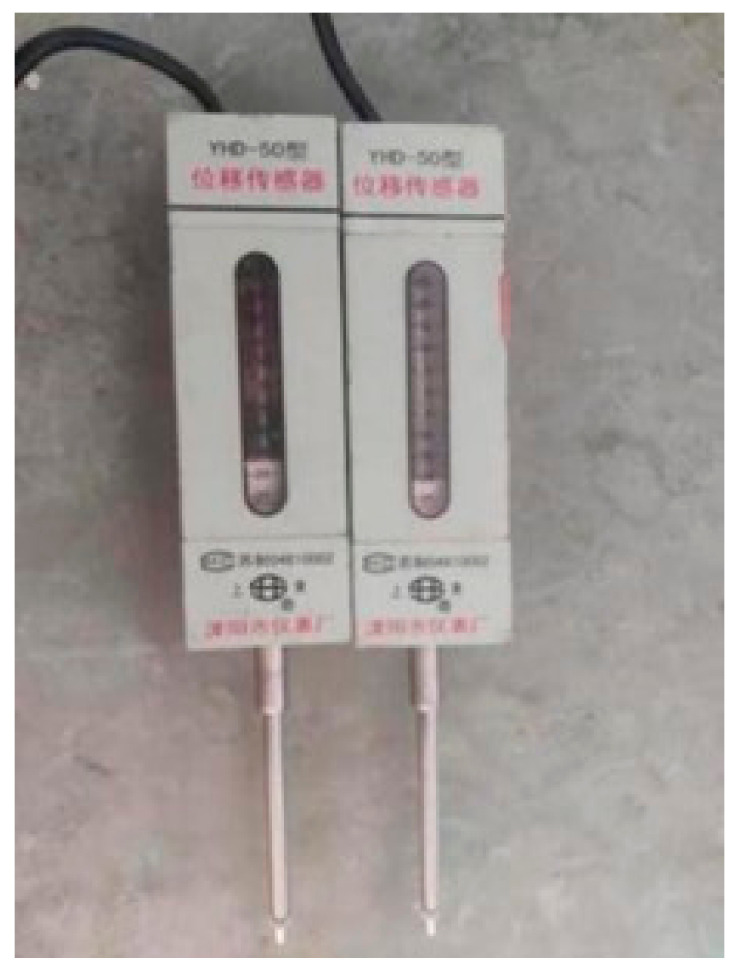
Displacement gauge.

**Figure 8:**
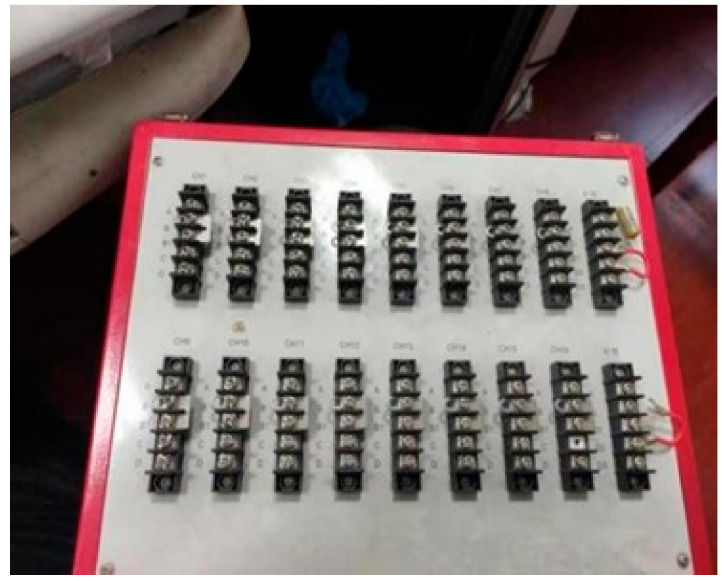
XL2101A16 Static Strain Gauge.

In this pull-out test, the universal testing machine utilizes displacement-controlled loading, with a loading rate set at 1 mm/min. When the hydraulic loading system is activated, the strain-acquisition system connected to the displacement gauge must simultaneously collect data. Under monotonic loading conditions, when the pull-out load decreases to 30% of the calibrated maximum load, loading should be halted, and the data should be preserved and recorded. The displacement gauge is shown in Figure 7. The strain acquisition instruments are shown in Figure 8.

## 3. Test Results and Analysis

### 3.1. Macro-Micro Deterioration Analysis Under Dry–Wet Carbonation Cycles

#### 3.1.1. Surface-State Changes

**Figure 9:**
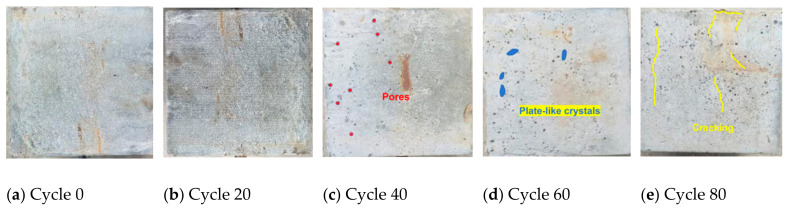
Surface Morphology of Reinforced Reef Limestone Concrete under Dry–Wet Carbonation Cycles.

Figure 9 shows the surface morphology of the reinforced reef limestone concrete specimens under dry-wet carbonation cycles. As seen in Figure 9, before 40 cycles of dry–wet carbonation, the surface changes of the specimens are not significant, with some damage observed on the surface and erosion peeling on the side of the reef limestone concrete. This may be attributed to insufficient vibration during the specimen casting process. These pores also provide channels for ion transport during carbonation cycles. As the number of dry–wet cycles gradually increases, fine cracks may appear on the specimen’s surface, and white flaky substances may precipitate. This could be due to the disappearance of moisture in the pores after experiencing dry–wet cycles, leaving behind crystalline ions. Due to the expansion of these ionic crystals, internal damage to the concrete may occur, leading to cracking of the concrete surface layer. These cracks and residual pores may provide pathways for the solution to re-enter the concrete during dry–wet cycles. Overall, these surface changes and erosion may result from the uneven structure of the specimen’s surface and the effects of moisture and salt during dry–wet cycles. This condition may exacerbate with the increasing number of cycles.

#### 3.1.2. Changes in Mass

An electronic balance with a precision of 0.1 g is used to measure mass, and the calculation formula for mass loss estimation is:

(5) $$W_{1}=(M_{t}-M_{0})/M_{0}$$

In the formula, W_1_ represents the mass loss rate, M_0_ is the initial mass of the specimen, and M_t_ is the mass of the specimen after corrosion for time t.

An ultrasonic testing instrument is used to characterize the damage after carbonation. The calculation formula for the elastic longitudinal wave velocity is:

(6) $$V_{p}=\frac{L}{t-t_{0}}$$

In the formula, V_p_ is the elastic longitudinal wave velocity of the specimen (m/s); L is the distance between the transmitting and receiving transducers at both ends of the specimen (m); t is the propagation time of the elastic longitudinal wave (s); and $t_{0}$ is the time-delay error of the ultrasonic testing system.

**Figure 10:**
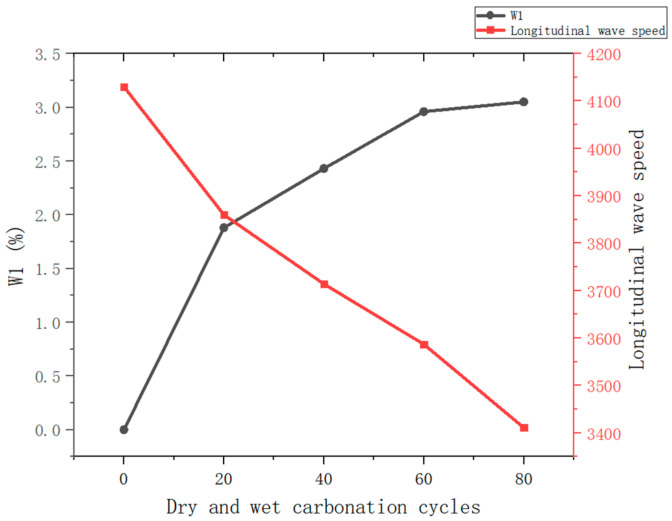
Changes in Mass Loss and Longitudinal Wave Velocity of Reef Limestone Concrete under Dry–Wet Carbonation Coupling Effects.

Figure 10 illustrates the changes in mass loss rate and longitudinal wave velocity of reinforced reef limestone concrete under dry–wet carbonation cycles. From the figure, it can be observed that as the number of dry–wet carbonation cycles increases, the mass loss of the reinforced reef limestone concrete shows a gradual increasing trend, while the longitudinal wave velocity exhibits a continuous decreasing trend. In the initial stage of dry–wet carbonation (0–20 cycles), the mass loss increases rapidly, mainly due to the alternation of dry and wet cycles causes the expansion and contraction of salt crystals in pores, exacerbating the propagation of microcracks. In the carbonation reaction, CO_2_ reacts with calcium hydroxide to form calcium carbonate, which shrinks in volume and reduces alkalinity, weakening the bonding structure. During the cycling process, cracks accelerate the penetration of CO_2_ and water, increasing the carbonation depth, while soluble salt precipitation and volume changes of carbonation products further damage the internal structure. The synergistic effect of physical expansion and chemical reaction leads to surface peeling. During this stage, mass loss increases from 0% to 1.88%.

With further increases in the number of dry–wet carbonation cycles (20–60 cycles), the mass loss stabilizes with a relatively slow growth rate. In the 60–80 cycle stage, the changes in mass loss tend towards a constant value, with the increase further diminishing, reaching 2.96% and 3.05%, respectively. Under the coupling effect of dry–wet carbonation, the ions enter into the pores of the reef limestone concrete, effectively filling the pore structure within the reinforced reef limestone concrete. In this process, the carbonation reaction continues, causing the reinforced reef limestone concrete to become denser. Hence, as the number of dry–wet carbonation cycles increases, there is a certain degree of increase in the mass loss of the reef limestone concrete.

After the number of dry–wet carbonation cycles exceeds 60, the increase in mass of the reinforced reef limestone concrete tends to stabilize. This phenomenon may be due to the penetrating ions forming crystals during drying, causing the internal pores of the concrete to expand into microcracks, exacerbating the internal damage of the reinforced reef limestone concrete. However, due to the minimal damage, the increase in the mass loss of the reinforced reef limestone concrete is not significant.

Furthermore, the longitudinal wave velocity can evaluate the changes in the pore structure related to internal damage within the concrete. The initial wave velocity of the reinforced reef limestone concrete is 4130 m/s. After 20 dry–wet carbonation cycles, the wave velocity reduces to 3860 m/s; after 40 cycles, it is 3714 m/s; after 60 cycles, 3587 m/s; and after 80 cycles, it decreases to 3411 m/s. This reduction in wave velocity may result from an increase in internal pore defects within the reef limestone concrete under dry–wet carbonation conditions, leading to a decrease in wave velocity as the number of dry–wet cycles increases. As the number of carbonation cycles increases, the longitudinal wave velocity declines rapidly, indicating that internal damage in the reinforced concrete accumulates with increasing carbonation cycles.

#### 3.1.3. Carbonation Mechanism of Concrete

After the concrete is poured, a hydration reaction occurs between the cement and water inside, which then combines with the coarse aggregates in the concrete to form a solid mass. Therefore, the hydration reaction is the process of the concrete gradually hardening. It also helps to maintain an alkaline environment within the concrete. The high pH environment can form a passivation layer that prevents the oxidation of the reinforcing bars and reduces the risk of rebar corrosion.

**Figure 11:**
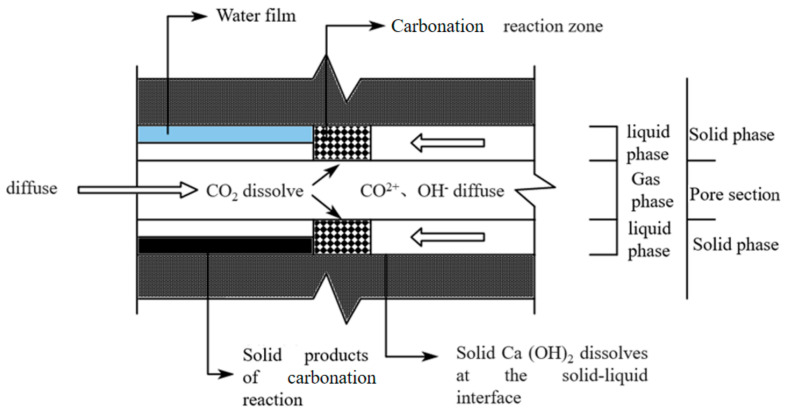
Principle Diagram of Concrete Carbonation.

However, during the hydration reaction of concrete, CO_2_ in the atmosphere can enter the interior of the concrete through the pores on its surface, where it dissolves in the pore solution. It then reacts with the products of the hydration reaction, Ca(OH)_2_ and CSH, to produce calcium carbonate. As this reaction progresses, the dissolved Ca(OH)_2_ in the pore water migrates towards the carbonation zone, leading to a gradual increase in carbonation depth. In this process, the pH value of concrete gradually decreases, causing corrosion of the reinforcing bars within the concrete. This leads to a reduction in the bond strength at the interface between the rebar and the concrete, decreasing the durability of the reinforced reef limestone concrete and ultimately resulting in member failure. The principle of concrete carbonation is illustrated in Figure 11.

### 3.2. Analysis of Bonding Performance of Reef Limestone Anchors Under Dry–Wet Carbonation Cycles

#### 3.2.1. Load-Slip Curve

**Figure 12:**
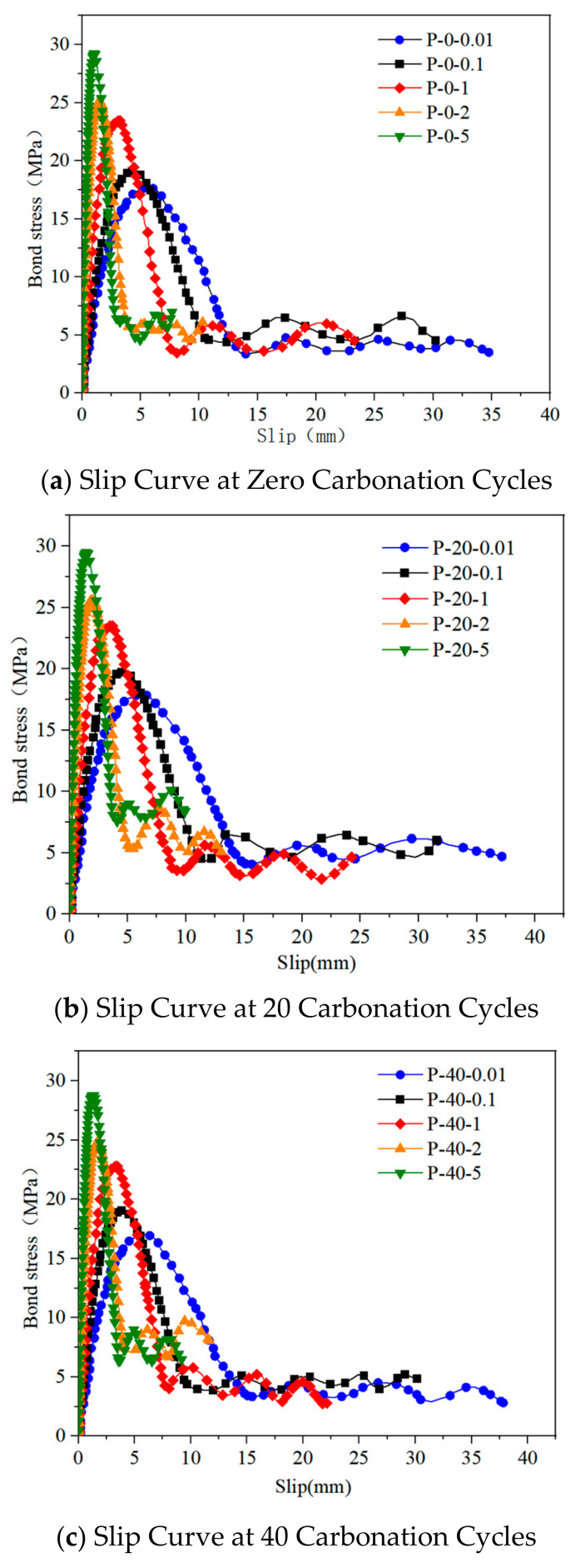

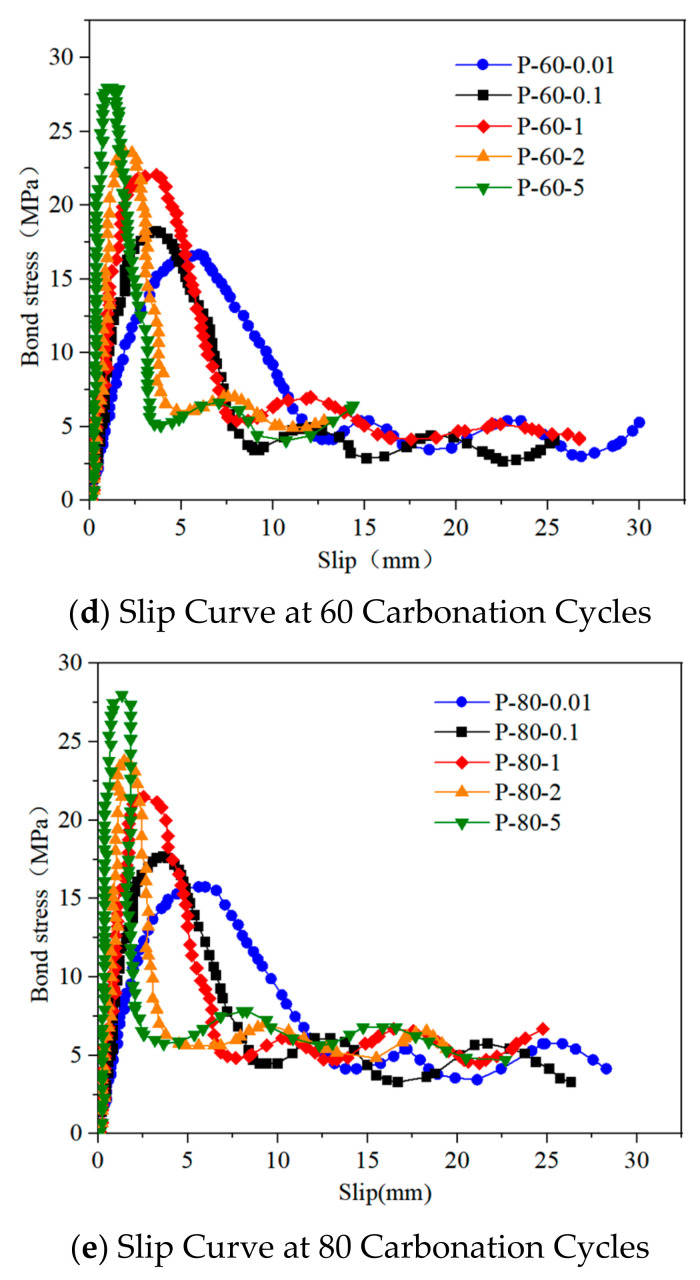
Bond slip curve of reinforced reef limestone concrete under different wet–dry carbonation cycles.

Research on the bonding performance of reinforced reef limestone concrete under different loading rates was conducted for 0, 20, 40, 60, and 80 cycles of dry–wet carbonation, and the bond-slip curves for each specimen are shown in Figure 12. The curves indicate that the effect of different loading rates on the failure of reinforced reef limestone concrete primarily involves changes in the peak bond stress.

From Figure 12, it can be seen that the bond-slip curve of the reinforced reef limestone concrete can be divided into four stages: the strength-increasing stage, the non-linear strength-increasing stage, the strength-decreasing stage, and the strength-stabilization stage, each characterized by significant differences in the evolution of bond strength and failure mechanisms.

The first stage (strength-increasing stage): In the early-loading phase, external forces are gradually transmitted through the rebar to the reef limestone concrete, during which there is no relative slip between the rebar and concrete, indicating a good bonding state. In this stage, the bond stress is primarily provided by mechanical adhesion, and the interface between the rebar and reef limestone concrete remains in a linear elastic working state. Since the initial loading force is small, localized stress concentration occurs near the loading end of the rebar, possibly resulting in slight peel-off in the local area. However, there is no slip at the free end, and the overall state remains fully bonded.

The second stage (non-linear strength-increasing stage): As the load increases, the bond stress gradually accumulates, leading to an increase in slip. At this point, the growth rate of the load is faster than the increase in slip, resulting in a non-linear upward trend in the bond-slip curve until it reaches the peak bond stress. Due to the high stiffness of the reef limestone concrete, the internal stress distribution during the load transfer process is uneven, making the failure process progressive. When the free end of the rebar begins to be affected and shows signs of damage, the interface’s mechanical adhesion forces gradually diminish. Additionally, the ribbed structure on the surface of the rebar can cause localized stress concentration in the concrete, making it easier for the concrete near the rebar to undergo tensile failure. It is noteworthy that under dry–wet carbonation cycles, the rebar may experience corrosion, reducing the bonding performance at the interface between the rebar and the reef limestone concrete. This degradation damage exhibits heterogeneity and randomness, further exacerbating the non-linear variation characteristics of bond strength. Therefore, in this stage, the anchorage of the reinforced reef limestone concrete demonstrates significant non-linear bond-failure characteristics.

The third stage (strength-decreasing stage): Once the load reaches its peak, the reinforced reef limestone concrete begins to enter the failure phase, and the bond strength gradually declines. At this point, as the slip increases, the bond stiffness significantly decreases, and the load gradually enters a declining range. The bond strength in this stage is primarily provided by friction and mechanical interlocking forces. However, as the rebar continues to slip, the concrete at the rebar ribs breaks sequentially along the interface, leading to a continual decrease in bond strength. Meanwhile, the contact interface between the rebar and reef limestone concrete continues to fracture, with some concrete being ground into fine powder, further weakening the bond strength and resulting in a trend of load decrease with increasing slip.

The fourth stage (strength-stabilization stage): When the slip of the rebar exceeds the rib spacing, the reef limestone concrete between the rebar ribs is completely sheared off, and the bond-slip curve enters a stable stage. At this point, the mechanical adhesion forces at the interface have completely disappeared, and the bond strength relies mainly on friction, gradually stabilizing as the slip increases. In the later stages, the particles at the contact interface between the reef limestone concrete and the rebar become extremely fine due to abrasion, leading the friction coefficient to approach a constant value. Consequently, the bond-slip curve exhibits a certain oscillatory trend and eventually reaches a stable residual bond-stress level.

Figure 12 illustrates the load-slip curves of reinforced limestone concrete under different wet–dry cycling counts. Compared to the peak bond strength of 30.54 MPa at zero cycles, when the number of cycles reaches 80, the peak bond strength significantly decreases to 27.46 MPa, a reduction of approximately 10%. Additionally, as the number of cycles increases, the slip amount after reaching the peak load gradually increases. The primary reason for this is the reduction in concrete pH caused by carbonation, which leads to the destruction of the passivation film on the reinforcement and the expansion of corrosion products that compress concrete, weakening the interfacial bond. At the same time, the ions’ precipitation fills the pores but later initiates the expansion of microcracks. The extension of the microcrack network and the deterioration of the interfacial transition zone (ITZ) reduce the interfacial friction resistance, making the slip process easier to sustain.

At the same wet–dry cycling counts, as the pulling rate increases, the peak bond strength also gradually rises. The main reason for this is that during rapid loading, the time for external force transmission at the reinforcement-concrete interface is shortened, leading to a more concentrated stress distribution and improved carrying efficiency of the interfacial bonding strength, which delays the initiation of slip. When the pulling rate in the tensile test is relatively fast, the rapid loading suppresses the viscous flow of the cement matrix [22], allowing the material to accumulate higher elastic strain energy before failure, resulting in an increase in peak bond strength.

#### 3.2.2. Analysis of Bond Strength Characteristics and Failure Modes

The bond stress of reinforced reef limestone concrete is calculated using the average-bond-stress method. This method employs the tensile force from the loading test system, along with the diameter and surface area of the rebar, with the calculation formula provided as (7).

(7) $$\tau =\frac{P}{\pi dl_{n}}$$

In the formula: τ is the average bond stress of the reinforced concrete; P is the applied load on the sample; d is the diameter of the pull-out rebar in mm; and ln is the bond length of the rebar in mm. The bond stress for each condition is calculated in triplicate, and the average bond stress is obtained using the method outlined in Formula (8).

(8) $$\bar{\tau }=1/3(\tau_{1}+\tau_{2}+\tau_{3})$$

The calculated results of the bond stress of reinforced reef limestone concrete under different dry–wet cycles are summarized in Table 6.

**Figure 13:**
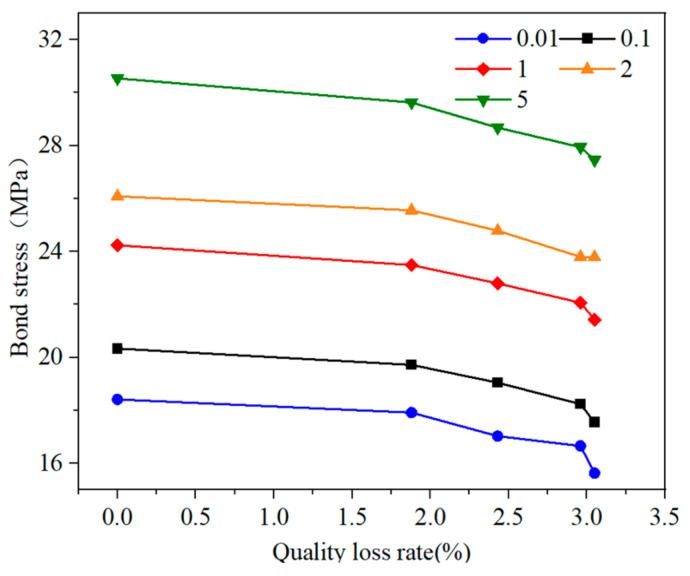
Relationship between bond strength and mass loss rate of reinforced reef limestone concrete at different rates.

Table 4 shows that the bond strength of the reinforced reef limestone concrete can reach 30.54 MPa when it is not carbonated, and the bond strength of the specimens increases with the increase in loading rate. When the number of dry–wet cycles increases to 80, the bond strength decreases to only 27.46 MPa. The number of dry–wet cycles negatively affects the bonding performance of the reinforced reef limestone concrete. The fitted curves of mass loss with loading rate and bond strength are shown in Figure 13.

From Figure 13, it can be seen that the impact of mass loss rate on bond stress is primarily significant after the mass loss reaches 2.5%. Prior to this point, the mass loss rate has a relatively small effect on bond stress. Once it exceeds 2.5%, the mass loss rate has a more pronounced impact on the bond stress of the reinforced reef limestone concrete. This can be analyzed as likely due to the fact that at lower dry–wet carbonation cycles, the effects on the specimens are confined to the surface without penetrating into the interior. As the number of cycles increases, the deterioration within the specimens becomes more evident, resulting in a larger decrease in bond stress.

**Figure 14:**
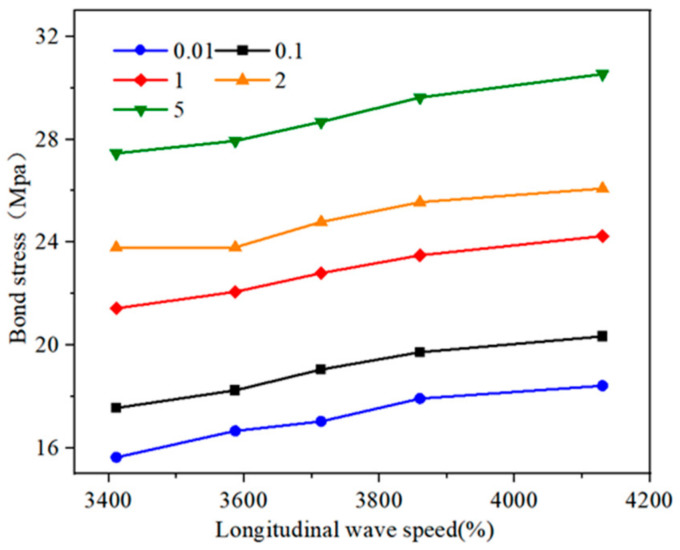
Relationship between bond strength and longitudinal wave velocity of reinforced reef limestone concrete at different rates.

The dry–wet carbonation cycles can cause internal damage to the reinforced reef limestone concrete. The longitudinal wave velocity can effectively characterize the damage state within the specimens. Therefore, the fitted curves of longitudinal wave velocity with loading rate and bond strength are shown in Figure 14.

#### 3.2.3. Failure Mode Analysis

Through experimentation, it was found that the failure mode of the bond interface in reinforced reef limestone concrete under different dry–wet carbonation cycles is predominantly splitting failure. When the number of dry–wet carbonation cycles is low, the cross-section is mainly split into two pieces. As the loading rate increases, the number of split sections also increases. Furthermore, with the increase in carbonation cycles, the peak load of the reinforced reef limestone concrete gradually decreases.

**Figure 15:**
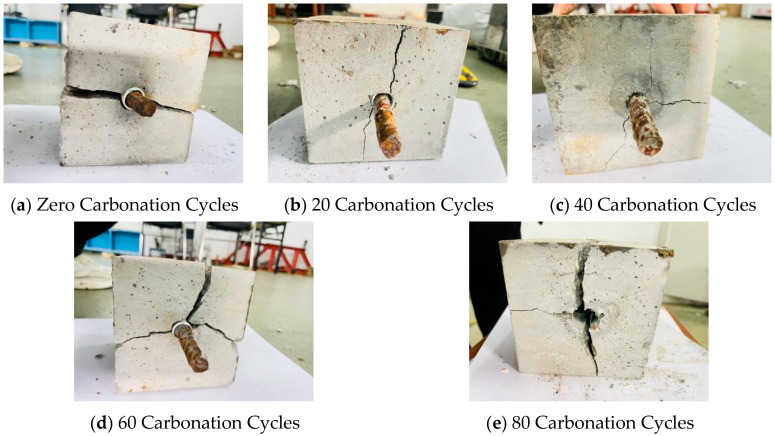
Bond Failure Modes at Different Carbonation Cycles.

From Figure 15, it can be observed that the main failure mode of the reinforced reef limestone concrete under carbonation conditions is splitting failure. Some concrete residues are present on the surface of the damaged rebar, and traces of ribs can be seen on the failure surface of the reef limestone concrete. The failure occurs with cracks developing along the diagonal of the specimen, while some cracks follow the centerline, indicating that the direction of splitting cracks is random. The splitting failure of the reinforced reef limestone concrete begins during the ascending phase of the bond-slip curve. As the number of dry–wet carbonation cycles increases, the number of split segments in the reinforced reef limestone concrete also increases. When there are 0 carbonation cycles, the splitting primarily occurs along the centerline. However, as the number of dry–wet carbonation cycles increases, weak points within the reef limestone concrete develop, leading to an increase in the number of splitting failures. When the number of dry–wet carbonation cycles reaches 40, the reinforced reef limestone concrete breaks into three pieces, and by 80 carbonation cycles, the failure morphology results in four pieces.

Analyzing the reasons, it is likely due to the increase in the number of dry–wet carbonation cycles, which facilitates the penetration of salts that crystallize and fill the pores inside the reinforced reef limestone concrete. As the number of dry–wet carbonation cycles continues to increase, the forces acting within the pores become amplified, leading to failure and creating weak planes within the reinforced reef limestone concrete, thereby increasing the extent of splitting failure.

## 4. Deterioration Model of Bond Slip for Reef Limestone Anchors Under Dry–Wet Carbonation Coupling Effects

By comparing the bond-slip strength of reinforced reef limestone concrete under different carbonation cycles and loading rates, bond-strength curves are plotted for varying dry–wet carbonation conditions. This analysis considers the effects of carbonation cycles and loading rates on the bonding characteristics of the concrete while also examining the bond characteristics influenced by these two factors.

**Figure 16:**
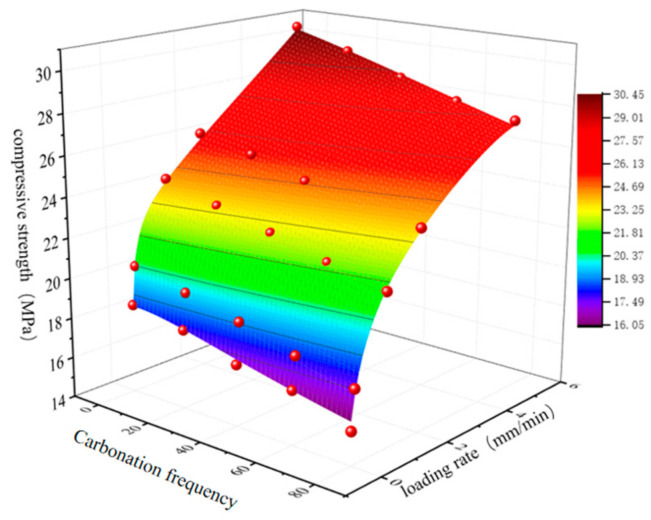
Bond Strength of Reinforced Reef Limestone Concrete.

From Figure 16, it can be observed that the bond strength of reinforced reef limestone concrete gradually increases with the increase in loading rate and gradually decreases with the increase in carbonation cycles. The bond strength of uncarbonated specimens is greater than that of carbonated specimens, indicating that dry–wet carbonation can cause damage to the bond interface in reinforced reef limestone concrete, thereby reducing the bond strength at the interface. This effect becomes increasingly pronounced with the increasing number of dry–wet carbonation cycles. Dry–wet carbonation can lead to rebar corrosion, resulting in decreased bonding performance. After the number of dry–wet carbonation cycles reaches 60, the reduction in bond strength compared to before 60 cycles becomes smaller, possibly because the reinforced reef limestone concrete has already fully carbonated by 60 cycles, making further increases in carbonation cycles have a lesser impact. Regarding the influence of loading rate, the number of dry–wet carbonation cycles affects bond strength. For the same loading rate, the number of dry–wet carbonation cycles experienced by the specimens causes a reduction in bond strength. As the loading rate increases, the bond strength of the specimens shows an increasing trend. The bond slip of reinforced reef limestone concrete follows the Rational 2D model, and the fitting equation is

(9) $$Z=\frac{\left(17.89+0.67x+205.2y-17.04y^{2}-1.78y^{3}\right)}{1+0.03x^{2}+1.13x^{2}-2.87x^{3}+8.7y+1.26y^{2}}$$

In the formula, x represents the number of dry–wet carbonation cycles, and y represents the loading rate of the tensile test.

Using this equation, the bond strength of reinforced reef limestone concrete can be calculated under the influence of other dry–wet carbonation cycle counts and loading rates, making it applicable to practical engineering.

## 5. Conclusions

This study systematically investigates the effects of dry–wet carbonation cycles and loading rates on the bond performance of reef limestone concrete through dry–wet carbonation cycling tests and pull-out tests. The following key conclusions were drawn:

(1) As the number of dry–wet carbonation cycles increases, the damage to reef limestone concrete intensifies progressively. Experimental results indicate that after 80 cycles, the mass loss rate of the concrete reached 3.05%, while the ultrasonic wave velocity decreased by 17.4%. These changes suggest that the carbonation cycles lead to damage in the internal pore structure of the concrete, which in turn affects its mechanical properties and durability.

(2) Reef limestone concrete exhibits significant differences in bond performance under various loading rates. Higher loading rates lead to higher peak bond strengths because rapid loading concentrates the external force on the interface between the rebar and concrete, thus improving the carrying capacity of the interfacial bond. Notably, at higher loading rates, the bond strength of the concrete is relatively higher, and the slip after peak load increases more slowly.

(3) The interaction between dry–wet carbonation cycles and loading rates has a complex effect on the bond strength of reef limestone concrete. As the number of dry–wet carbonation cycles increases, the bond strength of the concrete gradually decreases, and at higher cycle counts, the carbonation layer penetrates deeper into the rebar, leading to rebar corrosion and further deterioration of bond performance. However, higher loading rates can somewhat delay this deterioration, improving the bond performance of concrete under carbonation conditions.

(4) The study established a fitting equation based on experimental data to describe the effects of dry–wet carbonation cycles and loading rates on the bond strength of reef limestone concrete. This provides a theoretical basis for durability analysis and the design of concrete structures, enabling predictions of bond performance under different environmental conditions, particularly under dry–wet carbonation cycles. The findings offer valuable insights for optimizing design in engineering practice.

## Figures and Tables

**Table 1:** Reef limestone concrete mix proportion (kg/m^3^).

Name	Ordinary Portland Cement	Coarse Aggregate	Fine Aggregate	Groundwater	Fly Ash	Slag	Crack-Resistant Water-Proofing Agent
Quantity	780	700	300	250	70	150	15

**Table 2:** Physical properties of reef limestone aggregate.

Coarse Aggregate	Bulk Density (kg/m^3^)	Apparent Density (kg/m^3^)	Porosity %	Moisture Content %	Water Absorption %
	873	1939.6	59	13.2	22.9
Fine Aggregate	Bulk Density (kg/m^3^)	Apparent Density (kg/m^3^)	Fineness Modulus	Moisture Content %	Water Absorption %
	1392	2698.5	2.5	2.9	3.7

**Table 3:** Reinforcement strength index.

Steel Bar Model	Yield Strength/MPa	Tensile Strength/MPa	Elongation/%	Elastic Modulus
HRB400	472	630	22.5	208

**Table 4:** Chemical composition (g/L) required for seawater.

NaCl	MgCl_2_	Na_2_SO_4_	CaCl_2_	KCl	NaHCO_3_	KBr
24.53	5.20	4.09	1.16	0.695	0.201	0.101

**Table 5:** Dry–Wet Carbonation Cycle Scheme.

Sample Number	Number of Immersions	Number of Dryings	Number of Carbonation Cycles	Number of Specimens
P-0	0	0	0	15
P-20	20	20	20	15
P-40	40	40	40	15
P-60	60	60	60	15
P-80	80	80	80	15

**Table 6:** Average Bond Stress and Failure Modes.

Specimen Number	Peak Average Bond Stress (MPa) $\tau_{1}$ $\tau_{2}$ $\tau_{3}$			Mean Value	Failure Mode
P-0-0.01	18.32	18.61	18.35	18.42	Concrete Splitting
P-0-0.1	21.56	21.78	17.68	20.34	
P-0-1	23.96	24.57	24.19	24.24	
P-0-2	26.15	26.38	25.76	26.09	
P-0-5	29.65	30.81	31.16	30.54	
P-20-0.01	18.05	17.87	17.84	17.92	Concrete Splitting
P-20-0.1	18.91	19.59	20.66	19.72	
P-20-1	22.57	23.94	23.96	23.49	
P-20-2	24.62	26.57	25.49	25.56	
P-20-5	28.84	29.82	30.23	29.63	
P-40-0.01	16.68	17.45	16.96	17.03	Concrete Splitting
P-40-0.1	19.11	18.73	19.31	19.05	
P-40-1	22.51	22.52	23.37	22.8	
P-40-2	24.83	24.75	24.79	24.79	
P-40-5	28.91	28.59	28.54	28.68	
P-60-0.01	16.00	17.02	16.96	16.66	Concrete Splitting
P-60-0.1	17.95	18.23	18.54	18.24	
P-60-1	21.81	21.98	22.42	22.07	
P-60-2	23.49	23.93	23.93	23.8	
P-60-5	28.06	27.91	27.85	27.94	
P-80-0.01	15.59	15.42	15.88	15.63	Concrete Splitting
P-80-0.1	16.99	17.75	17.91	17.55	
P-80-1	21.53	21.09	21.67	21.43	
P-80-2	23.52	24.09	23.76	23.79	
P-80-5	26.61	28.13	27.64	27.46	

## Data Availability

The datasets presented in this article are not readily available because the data are part of an ongoing study or due to technical. Requests to access the datasets should be directed to 335257@whut.edu.cn.
